# Epidemiological Properties of Primary Open Angle Glaucoma in Nigeria

**DOI:** 10.1155/2013/402739

**Published:** 2013-05-26

**Authors:** Lawan Abdu

**Affiliations:** Department of Ophthalmology, Aminu Kano Teaching Hospital, Faculty of Medicine, Bayero University, PMB 3452, Kano, Nigeria

## Abstract

*Background*. Primary open angle glaucoma (POAG) is progressive chronic optic neuropathy in adults in which intraocular pressure (IOP) and other currently unknown factors contribute to damage. POAG is the second commonest cause of avoidable blindness in Nigeria. *Pattern of Presentation*. POAG is characterized by late presentation. Absence of pain which is a driving force for seeking medical help, inadequacy of trained eye care personnel, paucity of facilities, misdistribution of resources, lack of awareness, poor education, and poverty may all contribute to this. Medical and surgical treatment options available are challenging and tasking. *Screening for Glaucoma*. Screening is the presumptive identification of unrecognized disease (POAG) by applying test(s) which can be applied rapidly. Such test(s) should be of high reliability, validity, yield, acceptable, and cost effective. The test should ideally be sensitive, specific, and efficient. It is difficult to select a suitable test that meets these criteria. Intraocular pressure (IOP) appears to be the easiest option. But, high IOP is not diagnostic nor does normal value exclude the disease. Health education is a possible strategy in early case detection and management. *Treatment of POAG*. Glaucoma treatment can either be medical or surgical (this includes laser). Considering unavailability, potency, cost, and long-term effects of medication, surgery (trabeculectomy) could be a better option. Laser trabeculoplasty is available in a few centers. Viscocanalostomy is not routinely performed. Patient education is vital to success as management is for life. *Conclusion*. POAG remains a cause of avoidable blindness in Nigeria. There is need for long-term strategy to identify patients early and institute prompt management. Improvement in training of eye care personnel and provision of up to date equipment is essential in achieving this goal.

## 1. Introduction

Primary open angle glaucoma (POAG) is progressive chronic optic neuropathy in adults in which intraocular pressure (IOP) and other currently unknown factors contribute to damage and in which in the absence of other identifiable causes, there is characteristic acquired atrophy of the optic nerve and loss retinal ganglion cells and their axons [1]. POAG has been associated with risk factors such as central corneal thickness, structure of the optic nerve head, age, genetic factors, race, and intraocular inflammation among others. The global estimate of people with glaucomatous optic neuropathy is 60 million, and 8.4 million are blind from the disease. The highest prevalence of primary open angle glaucoma (POAG) occurs in Africans [2]. Various community-based studies have indicated that glaucoma is one of the leading causes of blindness in different parts of Nigeria. A study in Dambatta district in the northwestern part of the country showed that it accounted for 15% of blindness and 7% of those visually impaired [3]. Years before then a survey team that screened 1563 people of Hausa/Fulani ethnic extraction discovered that 1.02% of those aged 45 years and above had glaucoma [4]. Similarly, a study conducted in the south-west zone showed glaucoma accounted for 11.1% of blindness [5]. Institution-based studies have also indicated the importance of glaucoma as a cause of blindness. A study of 1794 workers in Ibadan confirmed glaucoma in 2.7% [6]. In Benin, 24.7% of 154 patients examined were blind from glaucoma based on visual acuity test, and the figure was higher when visual field criteria were used to define blindness [7]. A study of 555 new patients confirmed uniocular blindness from glaucoma in 6% [8]. More recently, the Nigerian National Blindness and Visual impairment Survey has shown that the cause specific prevalence of blindness due to glaucoma was 0.7% second only to cataract with 1.8%, and glaucoma blindness covers all the six geopolitical zones of the country though slightly higher in the south eastern part of the country. Glaucoma was the second commonest cause of blindness accounting for 16.7% [9]. Considering the challenge of identification and management of glaucoma on one hand and the deficit in human and material resources on the other hand, there is huge responsibility for caring of those who have glaucoma in Nigeria. That glaucoma is a common cause of visual disability in other countries of the west African region is not surprising perhaps due to sociodemographic similarities of the people. A cross-sectional survey in Akwapim-South district of Ghana showed glaucoma occurred in 7.7% of those above the age of 30 years and 8.5% of those aged 40 years and above [10]. A study in Togo showed that glaucoma was the second commonest cause of blindness with cause specific prevalence of 1.90% [11]. In the Cameroun, glaucoma accounted for 8% of 1343 blind patients seen in that hospital over a ten-year period [12]. 

## 2. Pattern of Presentation of POAG

The subtle nature in the initial stage of the disease, absence of pain which is a driving force for seeking medical help, inadequacy of trained eye care personnel, paucity of facilities offering eye care, misdistribution of resources for eye care (more than half of population is rural [13] and almost all eye care resources are located in the urban areas), and lack of awareness, poor education, and poverty may all contribute to the pattern of presentation of patients with glaucoma. Unlike in developed countries where health information is available to individuals, and diseases that occur in families are recorded, with increased level of education and awareness, the pattern of presentation is different from that in our environment. More often than not, patients present with advance disease with sight loss and massive optic nerve damage. A study showed that only 19% of patients had normal vision (6/6 to 6/18) at the time of presentation, and the remaining were either blind or severely visually impaired [14]. Another study reported that out of 98 patients, 30 were blind by visual acuity testing, and 45 were blind using field loss criteria [15]. The pattern may not be too different in other parts of Africa. In Ghana, only 17% had visual acuity better than 6/18 at presentation [16], while in Tanzania 41% had normal vision, 30% were visually impaired, and 29% were blind at presentation [17]. Early signs such as seeing haloes, rainbows, and intermittent clouding are often ignored by patients. In some instances enlightened patients can seek medical assistance due to reading difficulties of features suggestive of refractive errors which only after detailed evaluation in appropriate eye centers is the actual underlying problem discovered. Opportunistic case detection occurs from assessment of patients coming to the clinic irrespective of their primary complaints. Adults seeking driving license must pass an eye test [18]; however, some patronize quacks who endorses the document without conducting the proper assessment. Thus an opportunity to identify cases of glaucoma is lost. This problem is due to poor implementation of legal sanction for violators. In the absence of orthodox quality eye care services particularly in the rural areas patients patronize traditional eye healers (TEH) who are known to offer services detrimental to the goal of all eye care stake holders. TEH offer concoctions that can be tagged as traditional eye medicine (TEM). The composition, safety, and sterility of these medications are at best suspect further complicating the presentation, as patients may develop painful corneal ulcers or painful blind eye necessitating enucleation [19]. There are minimal difficulties in evaluating glaucoma patients in tertiary institution as most of the basic facilities are available. Optic nerve damage causes apparent papillary defect that can be detected by doing swinging light test. Optic disc evaluation with direct ophthalmoscope is routinely performed in addition to use of +90D, +60D with the slit lamp and other suitable lenses. Classical disc cupping is easily illustrated as many patients present with high cup: disc ratios, although a population study in a west African country showed that 3% of young adults had c : d ratio of 0.7 or higher suggesting high prevalence of glaucoma [20]. Anterior chamber angle is assessed with standard Goldmann goniolens. Studies have shown patients to have wide open angles (Shaffer grade III and IV) in all the patients examined and in 37.8%, respectively [14, 21]. High intraocular pressures (above 30 mmHg) by applanation tonometry are not unusual at presentation [14, 17, 22]. Visual field testing in yester years indicating classical changes such as peripheral depression, nasal step, temporal wedge, and massive peripheral visual constriction often using manual perimeters has largely been replaced by automated perimeters that show mean and pattern standard deviation and other reliability parameters. The tests are recorded, reproducible and amenable to comparison with further test made at a later date. Few studies have indicated field loss in patients with POAG, although one report showed field loss to within 30° of fixation in 48% of the patients studied [14]. The introduction of Stratus OCT machine in some tertiary centers has given additional capacity to diagnose, assess, and follow up patients with glaucoma. Retinal nerve fiber layer (RNFL) measurement is available, this greatly aid in objectively assessing the disease at presentation and monitoring stabilization or progression over time. RNFL measurements using OCT is important in making diagnosis of glaucoma and determines extent of ganglion cell loss at presentation in addition to monitoring progression of the disease. The advantages include the fact that it is a noncontact noninvasive procedure that produce in vivo retinal image and do not require pupillary dilation. Data obtained is stored in the system and can be retrieved and compared with that obtained at a later date. The machine is expensive for the health budget of most developing countries. Stratus OCT has lower acquisition speed, less depth resolution and has no 3-dimentional imaging technology like the latest Fourier Domain OCT. Studies to determine reference values have been conducted and about to be published. Some tertiary centers have equipment to do pachymetry and can more objectively determine the patient's intraocular pressure. Africans are known to have thinner cornea of about 534 *μ*m [23], and studies have suggested this as an independent risk factor for glaucoma [24].

## 3. The Challenge of Screening for Glaucoma

Screening is the presumptive identification of unrecognized disease or defect by applying test or examination or other procedures which can be applied rapidly. Such tests sort out apparently well person who probably have the disease from those who probably do not. As screening is not aimed to be diagnostic, persons with positive or suspicious findings are referred to physician for diagnosis and appropriate treatment [25]. One wonders which test can be applied to screen for glaucoma. Such test(s) should be of high reliability, validity, yield, acceptable, and cost effective. The test should ideally be sensitive, specific, and efficient. Among the various parameters, intraocular pressure (IOP) appears to be an easy option. But, is high pressure diagnostic and pathognomonic? There are people with high pressures having normal optic discs and fields, and others with abnormal discs and fields with IOP within “normal values.” This test requires skill and equipment. There is need to define person to screen. If age is considered it may be ideal to test from as young as twenties as a report from Benin showed high IOP levels in younger adults [26]. Index cases can be used to identify and screen first degree relatives [27]. Cup to disc ratios can be assessed although some studies have shown high ratios with normal IOP [28]. Employee screening can be employed to detect cases early [29]. Oculo-kinetic perimetry can be used as a screening test for glaucoma and has shown sensitivity of 94.7%, specificity of 98%, and efficiency of 92.6% in one report [30]. The difficulty is that none of the test can easily be applied on a large scale or at community level. Considering the principles of early disease detection criteria by Wilson and Jungner [25], glaucoma is of public health significance, there are facilities for diagnosis and treatment, and there are various treatment modalities for those recognized to have the disease. However, the latent stage may not easily be recognized. There is no single screening test that can be identified as suitable to the population. In most case one can identify cases to treat, though sometimes this is not so. The natural history of glaucoma cannot be said to be largely understood. Glaucoma is known to have ethnic predilection for west African, Afro-Caribbean, and those of Hispanic origin [31–34]. POAG with mutations in myocilin is said to be similar in phenotype; however, a study showed that mutation in myocilin has limited role in the pathogenesis [35]. A study in Ghana showed no statistically significant difference in mitochondrial DNA group between POAG and matched controls in the cohort [36]. Similarly, coding variant in optineurin may not contribute to risk of glaucoma in persons with west African descent [37]. Resources for genetic screening may therefore be counterproductive. Case finding is a continuous process in glaucoma; however, case finding may not be economically viable. Screening is a great challenge in low economies such as Nigeria.

## 4. Treatment of POAG

Treating glaucoma is mandatory, as the disease has been shown to reduce quality of life even in the early stages, and these worsen with increased severity [38]. The aim of treatment is to reduce IOP, although it is not the only risk factor for progressive optic nerve damage. IOP is, however, the risk factor most amenable to intervention. Glaucoma treatment can broadly be divided into two, namely, medical and surgical (this includes laser). Although the old concept of using “maximally tolerable” doses of medication has largely been abolished as a precondition for offering surgery, the current reality is more in favor of educating the patient about the disease and discussing the treatment options giving the advantages or otherwise of each alternative, so that the patient can make an informed decision. The reality is that even patients on whom decision is made to do surgery will require initial medication to reduce the pressure asidesandthe outcome of surgery may necessitates use of medication perhaps at a lesser intensity. There are various categories of antiglaucoma drugs such as the adrenergics (agonist and antagonist), miotics, carbonic anhydrate inhibitors (systemic and topical), and prostaglandins analogues to mention a few. They have individual and collective advantages and disadvantages. Medical treatment involves educating the patient about the use of medication for life. This brings the issue of compliance a great difficulty when factors such as age, cost, availability, and in our setting the prevalence of fake, substandard, adulterated, and expired (but relabeled/repackaged) drugs. Drugs including eye preparations are often stored in unventilated and hot storage areas and sold in measures-like grains. The potency and efficacy of such medications is suspect. Antiglaucoma medications are costly often only available in urban areas far away from majority of patients who are rural dwellers [13]. The level of poverty is so high that medicines can hardly compete with food at family level. A study of 120 consecutive glaucoma patients (46.7% of who were in the public service) showed a monthly cost of treatment of 105.4 USD [39] (with the national minimum wage at 112.5 USD per month). The cost of medical treatment is exorbitant. Short and long term side effects of medication are deterrence to compliance. One of the popular glaucoma drugs has been associated with tear film instability, and this can reduce compliance [40]. Among other factors, glaucoma patients using 2 or more eye drops have been shown to drop out of clinic [41]. There seems to be hope for medical treatment of POAG using a drug extracted from a locally available plant *Garcinia kola 0.5%* preparation which has demonstrated better mean IOP control (than a popular beta blocker) over a 24-week period, and the difference was statistically significant [42]. Having considered the various factors including the fact that 64.4% of Nigerian live below the income poverty line [43] and health insurance only covers regular sector of the workforce and not accessible to the poor. There is a need to consider the option of surgical treatment of patients with glaucoma. The success rate of trabeculectomy is less in black patients (67% compared to 80% in Caucasians), though the difference was not statistically significant [44]. Because of higher rate of failure of filtering procedure in dark races antifibrotic agents are routinely used during surgery [45]. The common types available are 5-fluorouracil (5FU) and mitomycin-C (MMC). Trabeculectomy is the most commonly performed type of glaucoma surgery. A report of the procedure performed on 139 eyes of 87 patients with preoperative IOP range 30–70 mmHg, showed postoperative IOP below 20 mmHg in 95.4% during 6–19 months follow-up period [46]. Similarly, a study of 71 eyes of 63 patients showed postoperative IOP of 10–15 mmHg in 82% of cases during follow up period [14]. A study of 46 patients who had the procedure with mean pre-operative IOP of 32.5 ± 6.2 mmHg showed reduction to 14.6 ± 4.2 and 13.5 ± 5.8 mmHg at 3 months and one year, respectively [47]. A study of 100 patients which defined success as IOP less than 22 mmHg or reduction of 30% below preoperative level reported success in 85%, 82%, and 71% at 1, 2, and 5 year postoperative period, respectively [48]. In a review of trabeculectomy that covered 433 eyes that had surgery over a 10-year period successful outcome was observed in 92% of patients with POAG [49]. The use of releasable sutures can further improve success rate of trabeculectomy with antifibrotic agent. A study showed that patients with mean preoperative IOP of 27.7 ± 5.9 mmHg had mean IOP after removal of the suture much lower than before removal (*P* > 0.0001) [50]. There is indication that MMC used is associated with lower likely hood of requiring postoperative medications and a greater likely hood of achieving IOP lowering without medications relative to use of 5FU in west African population, though the relationship is not statistically significant [51]. Most studies have shown minimal postoperative complications [14, 46–48, 50, 51]. A few reports showed reservations about long term results [52]. The success rate with other penetrating procedure such as deep sclerectomy is said to be low [53]. Viscocanalostomy, a procedure aimed at circumventing bleb-related complications [54, 55], is not commonly performed in our environment. There are virtually no reports of outcome of viscocanalostomy in Nigerian patients. In South Africa, a study of 60 randomly selected eyes of 60 patients who had viscocanalostomy with a mean preoperative IOP of 45.0 ± 12.1 mmHg reported a mean postoperative IOP of 15.4 ± 5.2 mmHg, 16.3 ± 4.2 mmHg, and 13.3 ± 1.7 mmHg at 12, 24, and 36 months, respectively [56]. The procedure has shown good tonometric results in glaucoma patients without complications of tracbeculectomy [57]. Aqueous shunts traditionally employed for medically uncontrollable glaucoma are not frequently used. A study of 24 eyes of 23 patients in South Africa in which Ex-PRESS miniature device was implanted under the sclera flap showed intraocular reduction from 27.2 ± 7.1 mmHg to 14.5 ± 5.0 at 12 months and 14.2 ± 4.2 mmHg at 24 months, respectively [58]. Some reports indicate that shunt procedure produces outcome comparable or higher than that of trabeculectomy [59, 60]. Argon Laser Trabeculoplasty (ALT) and Selective Laser Trabeculoplasty (SLT) are performed in a few centers. A definitive report on short and long term outcomes in Nigerian patients is awaited. Increasing glaucoma awareness and patient education is essential in reducing blindness from this disease. More community-based eye care workers need to be trained to identify those suspected to have glaucoma and refer to further evaluation. Government should make adequate precision in the health budget to cater for vulnerable groups such as those blinded from avoidable causes. Health insurance needs to be restructured to accommodate all citizens irrespective of their job status. A simple and systematic approach in examination will improve accurate diagnosis of glaucoma [61] and provide basis for appropriate management. Preventing glaucoma blindness in low income economy is a hard task, but it is never late to start.
